# Live Bacteria Supplementation as Probiotic for Managing Fishy, Odorous Vaginal Discharge Disease of Bacterial Vaginosis: An Alternative Treatment Option?

**DOI:** 10.7759/cureus.12362

**Published:** 2020-12-29

**Authors:** Lubna Mohammed, Moiz Javed, Aldanah Althwanay, Farah Ahsan, Federico Oliveri, Harshit K Goud, Zainab Mehkari, Ian H Rutkofsky

**Affiliations:** 1 Internal Medicine, California Institute of Behavioral Neurosciences & Psychology, Fairfield, USA; 2 Cardiology, California Institute of Behavioral Neurosciences & Psychology, Fairfield, USA; 3 Internal Medicine, California Institute of Behavioural Neurosciences & Psychology, Fairfield, USA; 4 Psychiatry, Neuroscience, California Institute of Behavioral Neurosciences & Psychology, Fairfield, USA

**Keywords:** vaginal health, probiotics, bacterial vaginosis, vaginal infections, lactobacillus, gardenella, probiotics in bacterial vaginosis, probiotics for vagina, live bacteria, recurrent bacterial vaginosis

## Abstract

Bacterial vaginosis (BV) is a universally prevalent cause of genital discomfort in females belonging to the reproductive age group, rendering the vagina more susceptible to various other complications. The standard treatment of BV involves using metronidazole and clindamycin, which help eliminate the infection but play no role in re-flourishing the normal vaginal homeostasis, which is lactobacilli preponderant, thereby rendering the vagina more prone to re-infection. Hence, clinical research has been performed to increase vaginal lactobacillus count through oral or vaginal supplementation. This current study's main objective is to review the previously conducted research regarding the efficiency of probiotic supplementation in the prevention and treatment of BV.

## Introduction and background

Bacterial vaginosis (BV) is one of the majorly prevalent gynecological diseases impacting 5%-58% of females belonging to the reproductive age group in various parts of the world [1]. It is also responsible for causing significant obstetric morbidity by affecting an average of 19.4% of pregnant women [2]. Although the incidence varies according to ethnicity, the highest recorded cases are amongst the African women, and the lowest case numbers belong to the regions of Asia and Europe. However, it is poorly understood how remarkably the cases differ within the ethnic classes belonging to the same country [1].

The syndrome mainly comprises an array of mucosal inflammation symptoms, discomfort causing fetid thin white/gray vaginal discharge, and burning and itching sensation in the vagina. However, there is a notable gross absence of exudation of leucocytes, rubor, and edema. Henceforth, it can be demarcated against the spectrum of vaginitis, so this was acknowledged as BV [2]. This disease has been related to a broad diversity of health complications over the past decades and is a major ongoing problem amongst females. Many cases remain asymptomatic as well, regardless of which there have been concerning associations reported of pelvic inflammatory disease and ensuing infertility [3], infections post-gynecological surgeries [4], and a rise in preterm births [2] in increasing incidences. Some studies have also demonstrated the link between this disease and the increased risk for human immunodeficiency virus (HIV) infection [1]. The clinical treatment of BV in the past decades has not shown any significant improvements. The management currently involves a standard therapy comprising the administration of antibiotics - clindamycin and metronidazole, which aid in killing the anaerobic pathogenic bacteria and repress their growth but do not aid in replenishing the typical vaginal symbiosis, making the vagina more prone to an unacceptably high recurrence rate of 40% to 50% in a year [5]. Furthermore, continued antibiotic subjection heightens the likelihood of the inception of resistant strains [6]. These facts relating to the disease's omnipresence and the recurrences make the research about any alternative supportive therapies an exigency to be used alongside antibiotics to improve the outcomes of this very frequently prevalent disease.

The normal vaginal flora mainly comprises Lactobacillus genus dominion. However, it can swiftly metamorphose into dysbacteriosis, where a plethora of micro-organisms can quickly increase in numbers, resulting in poly-microbial BV [7]. As Lactobacillus species (sp.) are the critical components of probiotics, they might have a role in treating BV because the Lactobacilli count of infected women shows significantly lower numbers as compared to robust females. Oral or intra-vaginal usage of these drugs is being scrutinized for their efficiency in subjugating the vagina and treating this disease or at least averting its repeated occurrences to reduce the female morbidity rate [8]. The use of probiotics, which are defined as "live micro-organisms, which, when administered in sufficient quantities, provide health benefits to the host" [9], are being considered as adjuvant therapy for BV. Research in the field of probiotics to alleviate diseases has a historical significance, as it had commenced as early as 1907 by Metchnikoff [10]. But after the creation of efficient antibiotics, which drastically changed the face of human disease and suffering, this field was soon forgotten to be studied. However, after the recent illustrations depicting the phenomena of how impactful the use of live bacteria can be in human health and disease, which was emphasized by the Human Microbiome Project in 2015, the number of clinical trials being conducted on the utility of probiotics for the treatment of various infections has shown a rapid spike [11]. They have been declared safe and highly effective supplementary therapy to treat antibiotic-associated diarrhea, as they help replenish the normal gut flora [12]. They are also being considered to replace antibiotics as a prophylactic measure for recurrent urinary tract infections [13]. This review article will discuss if these live lactobacilli-containing drugs can aid in the expedition of regaining typical vaginal homeostasis and help in prophylaxis and recovery from this most common vaginal discharge causing disease.

Search strategy

Detailed research was conducted using the keywords mentioned in Table 1 to recognize the studies analyzing and assessing the impact of live lactobacillus sp. as probiotics on BV's disease outcomes using PubMed as the primary database. Apart from the study's primary aim, BV community development's pathophysiology and the current treatment guidelines have also been included in detail in this study. All the articles taken into consideration were chosen without the restriction of time of publication or study type, i.e., traditional reviews, systematic reviews, clinical trials, case-control, and cohort studies. Studies were not refined based on age and ethnicity. There were no demographical limitations in the search. All the articles chosen were in the English language except one article used in this study that was originally in the Polish language, but a translated version was available on the PubMed database. As this is a traditional review article, Preferred Reporting Items for Systematic Reviews and Meta-Analyses (PRISMA) guidelines were not followed. Data were collected from inception up to November 2020 and are summarized in Table 1.

**Table 1:** Keywords and their search results BV = Bacterial Vaginosis

Keywords	Database	Number of results
Lactobacillus	PubMed	41,259 results
Probiotics	PubMed	30,169 results
Bacterial Vaginosis	PubMed	4,810 results
Gardnerella Vaginalis	PubMed	1,755 results
BV and Lactobacillus	PubMed	901 results
Recurrent Bacterial vaginosis	PubMed	497 results
Probiotics in Bacterial vaginosis	PubMed	318 results

## Review

Discussion

This section will discuss BV environment production's pathophysiology, various diagnosis criteria, and the current standard therapy for BV treatment. Various clinical trials demonstrating the use of probiotics in BV cure, recurrences, and ability to regain vaginal homeostasis has also been summarized.

The changing dynamics of the vaginal microbiome during BV

**Figure 1:**
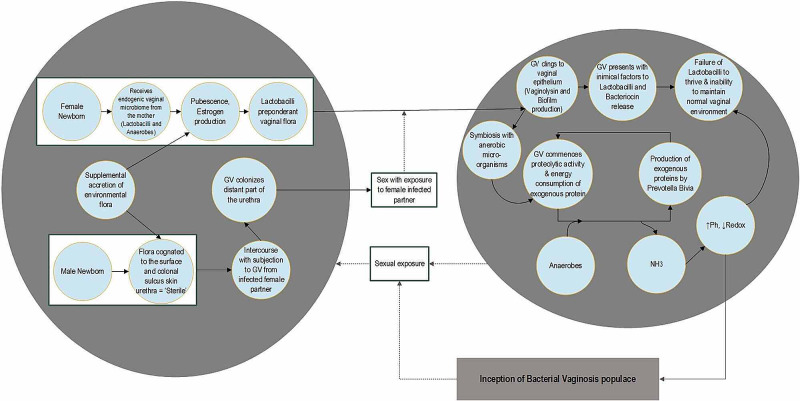
Descriptive Model of BV Community Development GV = Gardenella Vaginalis; NH3 = Ammonia; BV = Bacterial Vaginosis

The vaginal microbiome is an active habitat that can differ throughout a woman's lifespan in relation to exogenous and endogenous elements such as age, pregnancy, pharmacological interventions, and urogenital infections [14]. The life-long prevalence of Lactobacilli sp. dominance in the female vagina has been proven by various molecular identification tools. But in cases of vaginal dysbiosis, the Lactobacilli count gets significantly decreased along with replacement by other pathogenic micro-organisms (Gardnerella vaginalis (GV), Prevotella sp., and Mobiluncus sp.) as seen in various vaginal infections. This micro-flora identification has been performed by both conventional and molecular identification techniques [15]. Lactobacillus jensenii, Lactobacillus iners, Lactobacillus crispatus, and Lactobacillus gasseri are the majorly prevalent lactobacilli sp. found in the female vagina. However, there is great variation noted in the sp. in relation to the demographic population [16]. The flowchart in Figure 1 is a conceptual model developed to demonstrate the development of BV [17].

Although the precise etiology of BV development is unclear, this illustration demonstrates that GV is not a standard component of the female vaginal microbiome obtained at birth. The likelihood of sexual transmission is one of the major factors responsible for acquiring it [17]. Venereal transmission is substantiated by the retrieval of GV from the penile urethra of males having intercourse with infected females. A significantly higher predisposition of relapse is noted in females, even after complete treatment due to having exposure to their infected male partner [18]. GV has vehement adherence properties to the female vaginal epithelium by utilizing biofilms, thereby offering a matrix for additional morbific micro-organisms to attach. Additionally, these biofilms act like a barrier and make the antibiotic drugs relatively impenetrable, making it more challenging to eliminate the infection [19].

Diagnosis of BV

The diagnosis of BV is confirmed by two main categories: clinical criteria and laboratory-based testing. The universally recognized clinical criteria are Amsel's criteria [20], which has a requirement of three out of four criteria to be fulfilled. Physicians can utilize this criterion for quicker office-based diagnosis and immediate commencement of treatment drugs.

Amsel’s Criteria

-Vaginal pH greater than 4.5

-Presence of clue cells

-Milky, homogenous vaginal discharge

 -Amine (fishy) odor after the addition of 10% KOH to the vaginal fluid

Nugent Scoring System

The laboratory diagnosis is highly sensitive and is called the Nugent scoring system, which is done through a gram-staining technique that involves scoring the identified vaginal microbes in the vaginal fluid. Lactobacillus sp. when identified, is given the score 0-4; GV sp., Bacteroides sp., Mobilincus sp. are marked a score of 4-6; abnormal microflora with vast numbers of anaerobic bacteria with the complete absence of lactobacilli is scored as 7-10 [21].

Other techniques involved in the confirmation of diagnosis, which is relatively uncommon in comparison to Amsel's criteria and the Nugent scoring system, are bacterial culture, molecular-based polymerase chain reaction assays, and microbiota analysis [22].

Other diseases that fall under the category of differential diagnosis of BV are vulvovaginal candidiasis and Trichomonas vaginitis. As BV infection renders the female very vulnerable to acquiring other sexually transmitted Infections, co-infection with Neisseria gonorrhea and Chlamydia trachomatis should also be taken under consideration before making a diagnosis. Furthermore, HIV testing should be suggested to women with a confirmed diagnosis of BV, as they are at heightened risk of acquisition of it in comparison to healthy females [23].

Treatment of BV

Current Treatment Guidelines Suggested by the Centres for Disease Control (CDC)

Any one of the following:

-Oral administration of metronidazole 500 mg tablets twice a day for one week

-Intravaginal application of one applicator of 0.75% metronidazole gel every night for the duration of five consecutive nights

-Intravaginal application of one applicator of 2% clindamycin vaginal cream for successive nights for one week

Alternative treatment options include any one of the following:

-Oral administration of tinidazole 2 g/day for two consecutive days

-Oral administration of tinidazole 1 g/day for five days

-Oral administration of clindamycin tablets twice daily for one week

-Intravaginal instillation of clindamycin 100 mg ovules for three consecutive nights

The treatment recommendations stay the same for females, either pregnant or non-pregnant. The suggestions for managing the recurrences are not universally uniform. However, the CDC has advised that to manage recurrence, it should be done by utilizing the same drugs used to treat the initial infection. Despite many theories proposing BV to be a sexually transmitted disease, there must be no treatment offered to the infected women's male sexual partners, as per the CDC treatment protocols [23].

Role of probiotic therapy

Probiotic drugs comprise living micro-organisms that positively impact the host's health when administered and are being encouraged as possible substitutes for prophylaxis and cure of various infections. Treatment of vaginal infections by probiotics is at the forefront of quickly evolving research fields [24].

Many earlier research pieces have proved the efficacy of probiotic supplementation in treating various vaginal infections like BV and Vulvovaginal candidiasis by causing a positive alteration in the vaginal microbiome [8]. The sp. generally utilized in probiotics manufacturing includes Lactobacillus, Enterococcus, Lactococcus, Streptococcus, and Bifidobacterium. But the most preferred strain that exerts the maximal benefits in vaginal repopulation is the Lactobacillus genus. Lactobacillus sp. is responsible for breaking down carbohydrates and maintaining the acidic vaginal environment by lactic acid and carbon dioxide production, thereby inhibiting the excessive growth of pathogenic microflora like Enterobacteria, Escherichia coli, Candida, and GV [25].

Some distinct features of Lactobacilli species that prevent morbific micro-organisms from colonizing the vagina are:

-Various kinds of secretions, bacteriocins, lactic acid, and hydrogen peroxide release causes the direct lethal effects of pathogenic micro-organisms.

-Co-aggregation capabilities amongst the lactobacilli and infectious micro-organisms cause them to be pushed distant from the vaginal epithelium, rendering them more prone to be affected by the secreted factors that have destructive properties.

-The lactobacillus species can directly compete with other micro-organisms for adhesion with host cell receptors in the vaginal epithelium, thereby preventing the pathogens from the inception of a pathogenic environment [24].

**Figure 2:**
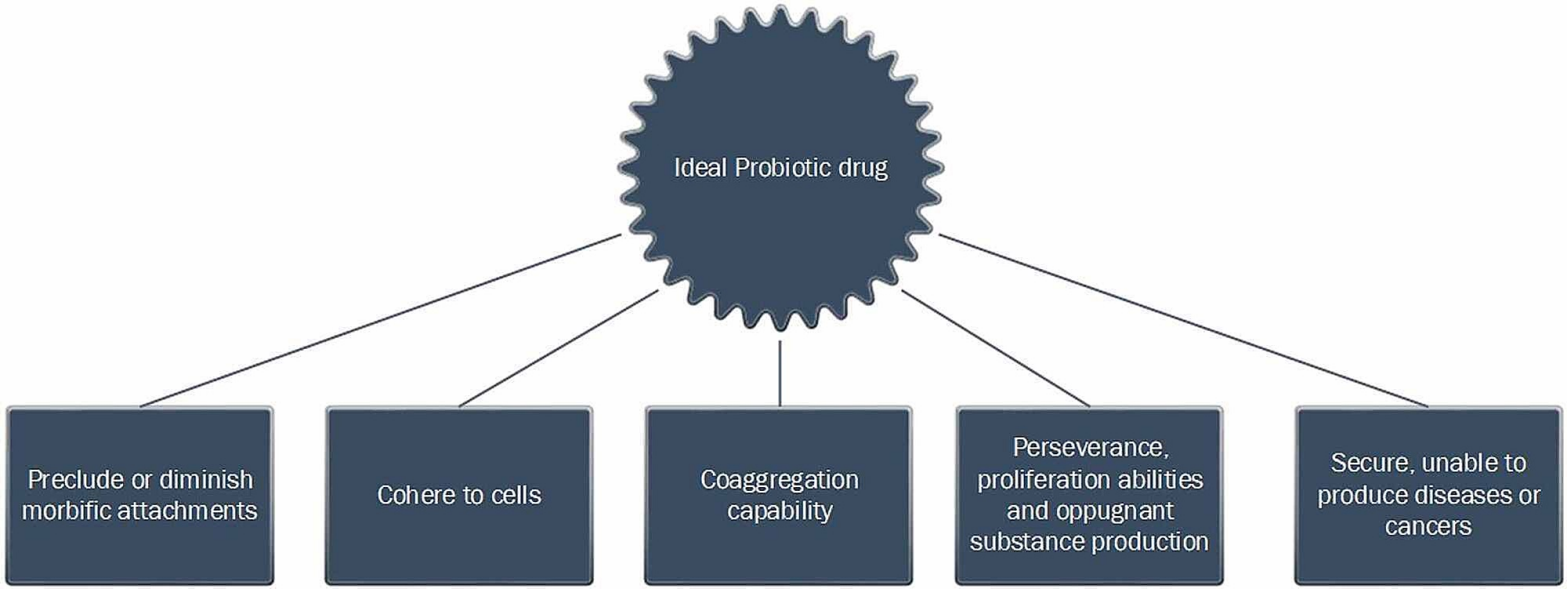
Features of an Ideal Probiotic Drug

Figure 2 demonstrates the features that should be present in a probiotic drug to be declared suitable for human consumption [26].

Clinical trials on probiotic drugs in BV

The efficacy of live lactobacillus strains in the management of BV and their abilities to restore the vaginal homeostasis in the vagina have been demonstrated by several clinical trials and are summarized in Table 2.

**Table 2:** Summary of clinical trials conducted on the use of probiotics in BV BV = Bacterial Vaginosis; RCT = Randomized Controlled Trial

Author	Study population	Study type	Probiotic used	Outcome in the control group
Vujic et al., 2013 [27]	544; Numbers (n) n=395 in probiotic, n= 149 in placebo.	Randomized Control Trial (RCT); double-blinded	Lactobacillus rhamnosus GR-1 and Lactobacillus reuteri RC-14	BV diagnosis confirmed otherwise healthy subjects were given one probiotic drug and an indistinguishable placebo drug that was administered for six weeks. Results depicted that 61.5% of women regaining normal healthy vaginal microbiome, and only 26.9% of women in the placebo category had restitution.
Bradshaw et al., 2012 [28]	450; n= 133 in probiotic n= 140 in clindamycin, n= 135 in placebo.	RCT	Gynoflor (Lactobacillus acidophilus KS400 + 0.03 mg estriol)	The one-month recurrence rate of BV showed a slight reduction following consumption of twelve days of gynoflor after Oral metronidazole therapy (9/133) in comparison to placebo (12/135). Still, vaginal clindamycin was superior (5/140).
Tomusiak A et al.,2015 [6]	376 enrolled; 160 randomized, n=86 in probiotic, n= 74 in placebo.	RCT; multi-centered, placebo-controlled	inVag (Lactobacillus fermentum 57A, Lactobacillus plantarum 57B, and Lactobacillus gasseri 57C)	After one week of intra-vaginal probiotic capsule administration, a notable reduction was observed in Nugent score and vaginal pH, along with a remarkable rise in the abundance of Lactobacillus numbers when counted in consecutive visits. Presence of specific strains from the probiotic administered, where noted in 82% of the women in the third visit.
Heczko et al., 2015 [29]	154; n = 73 in probiotic, n = 81 in placebo.	RCT	Lactobacillus fermentum 57A, Lactobacillus. plantarum 57B, and Lactobacillus gasseri 57C along with metronidazole for ten days + 10 days only probiotic/placebo follow-up	The recurrence period of BV symptoms was prolonged, up to 51% (p < 0.05) in contrast to the placebo. Microbiological parameters showed improvement seen by Lactobacillus count rising along with reduction and maintenance of low pH levels. Improvement in clinical symptoms was noted.
Wang Ya D et al., 2010 [30]	120; n= 58 in probiotic, n= 62 in placebo.	RCT	Lactobacillus rhamnosus, Lactobacillus acidophilus, and Streptococcus thermophiles for one week, then no intervention for a week followed up by last week of drug use.	Recurrence rates of BV showed a significant reduction of 9/57 in the treatment group compared to the placebo group of 27/60 women. Only 2/57 women showed the presence of GV, whereas 11/60 showed it after two months.
Zongxin Ling et al., 2012 [31]	115; n=30 in BV control cases, n=30 in healthy, n=30 in one-week metronidazole treated BV cases, n=25 BV positive cases using 10-day.	RCT	Lactobacillus crispatus DM8909	Cure rates of BV were checked after the 5^th^ day and after a month in follow-up visits revealing the probiotic group performing better (88% and 96% respectively) in comparison to the metronidazole using group (83.3% and 70% respectively).
Ehrstorm et al., 2010 [32]	95; n=60 in probiotic group, n=35 in placebo.	RCT	Lactobacillus gasseri LN40, Lactobacillus fermentum LN99, Lactobacillus casei subsp. Rhamnosus LN113 and Pediococcus acidilactici LN23, for five successive days	The intervention group endured lesser malodorous discharge and showed clinical improvement in 2-3 days. Lactobacilli strain colonization was also proven to be promising in the probiotic group 89% (47/53) compared to 0% of the placebo group in the second visit.
Russo et al., 2018 [33]	40; n=20 in probiotic n=20 in placebo.	RCT	Oral Lactobacillus acidophilus GLA-14 and Lactobacillus rhamnosus HN001 mixture, along with Lactoferrin for 15 days.	Oral supplementation resulted in vaginal colonization of the Lactobacillus strains along with significant improvement in symptoms of discharge, odor, and itching. Nugent score also showed re-establishment of normal microbiome.

One of the trials did not instill the probiotic during menstrual cycles, and it was only offered peri-menstrually [29], all the remaining studies included probiotics regardless of the menstrual status. The results of the studies did not fluctuate significantly and gave a generally positive consensus. A study also demonstrated that antibiotic therapy was more beneficial in BV infection elimination in comparison to only probiotics [28].

Many studies suggested that exogenous Lactobacilli sp. supplementation leads to a quicker shift back to the vaginal homeostasis from the disease state [6,27,29,31-32]. It was also proved that orally instilled Lactobacilli sp. were capable of colonizing the vagina [33]. The exogenously administered Lactobacilli species' ability to reduce the vaginal pH to maintain a favorable acidic environment was also depicted by a noticeable fall in pH after the administration of probiotics [28]. It was also demonstrated that the instillation of probiotic drugs was useful in many subjects, reflected by significantly improved clinical outcomes by reducing malodorous discharge and itching [6,29,32-33], and covered the reduction in Nugent grades, which is a reflective factor of BV treatment. Studies also illustrated the dramatic reduction in the recurrence rates of BV post-probiotic therapy administration [28-30].

Limitations

This study has several limitations because the studies included in this paper had relatively smaller study groups, so the findings cannot be generalized ubiquitously to the entire population. The probiotic drugs contained a 'cocktail' of Lactobacillus sp. strains along with additional other drugs (metronidazole, estriol, non-Lactobacilli probiotic strains) in some studies, which makes it difficult for accurate outcome evaluation. Differentiation amongst Lactobacilli sp. colonized either from probiotics or host endogenous Lactobacilli sp. was not possible using the traditional diagnostic criteria. The Lactobacilli sp. counts may be falsely increased if the samples are taken soon after probiotic insertion. Even though the studies in this paper reflected that the usage of probiotics in BV is plausible, data evaluating clear and consistent evidence regarding the positive impact of probiotics in female vaginal health is still lacking.

## Conclusions

It was proved that the BV community is established due to a reduction in the numbers of Lactobacilli, thereby allowing morbific GV to pioneer. The efficacy of administration of exogenous and endogenous Lactobacilli supplementation in the form of probiotics was studied in the treatment of BV. It was demonstrated that probiotics have a promising scope in reducing vaginal pH, restoring normal vaginal flora, and reducing recurrences in the limited study population. Extensive research is the dire need of the hour in this field. Suggestions for future research include: as heterogeneity is noted in the vaginal microbiome composition demographically, trials with subjects belonging to a plethora of regions should be conducted; treatment should be offered with only one Lactobacilli sp. at a time to narrow down the most beneficial strain; and the study population should include a larger number of subjects, as all the clinical trials that have been conducted to date have comprised small study groups.

## References

[1] Kenyon C, Colebunders R, Crucitti T (2013). The global epidemiology of bacterial vaginosis: a systematic review. Am J Obstet Gynecol.

[2] Onderdonk AB, Delaney ML, Fichorova RN (2016). The human microbiome during Bacterial Vaginosis. Clin Microbiol Rev.

[3] Ness R B, Kip K, Hillier S (2005). A cluster analysis of bacterial vaginosis-associated microflora and pelvic inflammatory disease. Am J Epidemiol.

[4] Larsson PG, Bergström M, Forsum U (2005). Bacterial vaginosis. Transmission, role in genital tract infection and pregnancy outcome: an enigma. APMIS.

[5] Balkus J, Srinivasan S, Anzala O (2017). Impact of periodic presumptive treatment for bacterial vaginosis on the vaginal microbiome among women participating in the Preventing Vaginal Infections Trial. J Infect Dis.

[6] Tomusiak A, Strus M, Heczko PB (2011). Antibiotic resistance of Gardnerella vaginalis isolated from cases of bacterial vaginosis [Article in Polish]. Ginekol Pol.

[7] Ma B, Forney LJ, Ravel J (2012). Vaginal microbiome: rethinking health and disease. Annu Rev Microbiol.

[8] Falagas M, Betsi GI, Athanasiou S (2007). Probiotics for the treatment of women with bacterial vaginosis. Clin Microbiol Infect.

[9] Reid G, Jass J, Sebulsky MT, McCormick JK (2003). Potential uses of probiotics in clinical practice. Clin Microbiol Rev.

[10] Wang Z, He Y, Zheng Y (2019). Probiotics for the treatment of bacterial vaginosis: a meta-analysis. Int J Environ Res Public Health.

[11] Sartor RB (2004). Therapeutic manipulation of the enteric microflora in inflammatory bowel diseases: antibiotics, probiotics, and prebiotics. Gastroenterology.

[12] Hempel S, Newberry SJ, Maher AR (2012). Probiotics for the prevention and treatment of antibiotic-associated diarrhea: a systematic review and meta-analysis. JAMA.

[13] Stapleton AE, Au-Yeung M, Hooton TM (2011). Randomized, placebo-controlled phase 2 trial of a Lactobacillus crispatus probiotic given intravaginally for prevention of recurrent urinary tract infection. Clin Infect Dis.

[14] Huang B, Fettweis J, Brooks J, Jefferson KK, Buck GA (2014). The changing landscape of the vaginal microbiome. Clin Lab Med.

[15] Stoyancheva GD, Danova ST, Boudakov IY (2006). Molecular identification of vaginal lactobacilli isolated from Bulgarian women. Antonie van Leeuwenhoek.

[16] Martínez-Peña MD, Castro-Escarpulli G, Aguilera-Arreola MG (2013). Lactobacillus species isolated from vaginal secretions of healthy and bacterial vaginosis-intermediate Mexican women: a prospective study. BMC Infect Dis.

[17] Muzny CA, Schwebke JR (2016). Pathogenesis of bacterial vaginosis: discussion of current hypotheses. J Infect Dis.

[18] Zozaya M, Ferris MJ, Siren JD (2016). Bacterial communities in penile skin, male urethra, and vaginas of heterosexual couples with and without bacterial vaginosis. Microbiome.

[19] Sobel JD (2015). Editorial commentary: vaginal biofilm: much ado about nothing, or a new therapeutic challenge?. Clin Infect Dis.

[20] Amsel R, Totten PA, Spiegel CA, Chen KCS, Eschenbach D, Holmes KK (1983). Nonspecific vaginitis. Diagnostic criteria and microbial and epidemiologic associations. Am J Med.

[21] Nugent RP, Krohn MA, Hillier SL (1991). Reliability of diagnosing bacterial vaginosis is improved by a standardized method of gram stain interpretation. J Clin Microbiol.

[22] Van den Munckhof E, van Sitter R L, Boers K E (2019). Comparison of Amsel criteria, Nugent score, culture and two CE-IVD marked quantitative real-time PCRs with microbiota analysis for the diagnosis of bacterial vaginosis. Eur J Clin Microbiol Infect Dis.

[23] Workowski KA, Bolan GA (2015). Sexually transmitted diseases treatment guidelines. MMWR Recomm Rep.

[24] Spurbeck RR, Arvidson CG (2011). Lactobacilli at the front line of defense against vaginally acquired infections. Future Microbiol.

[25] Kim JM, Park YJ (2017). Probiotics in the prevention and treatment of postmenopausal vaginal infections: review article. J Menopausal Med.

[26] Borges S, Silva J, Teixeira P (2014). The role of lactobacilli and probiotics in maintaining vaginal health. Arch Gynecol Obstet.

[27] Vujic G, Knez A, Stefanovic V, Vrbanovic VK (2013). Efficacy of orally applied probiotic capsules for bacterial vaginosis and other vaginal infections: a double-blind, randomized, placebo-controlled study. Eur J Obstet Gynecol Reprod Biol.

[28] Bradshaw CS, Pirotta M, De Guingand D (2012). Efficacy of oral metronidazole with vaginal clindamycin or vaginal probiotic for bacterial vaginosis: randomised placebo-controlled double-blind trial. PloS One.

[29] Heczko PB, Tomusiak A, Adamski P (2015). Supplementation of standard antibiotic therapy with oral probiotics for bacterial vaginosis and aerobic vaginitis: a randomised, double-blind, placebo-controlled trial. BMC Women's Health.

[30] Ya W, Reifer C, Miller LE (2010). Efficacy of vaginal probiotic capsules for recurrent bacterial vaginosis: a double-blind, randomized, placebo-controlled study. Am J Obstet Gynecol.

[31] Ling Z, Liu X, Chen W (2013). The restoration of the vaginal microbiota after treatment for bacterial vaginosis with metronidazole or probiotics. Microb Ecol.

[32] Ehrström S, Daroczy K, Rylander E (2010). Lactic acid bacteria colonization and clinical outcome after probiotic supplementation in conventionally treated bacterial vaginosis and vulvovaginal candidiasis. Microbes Infect.

[33] Russo R, Edu A, De Seta F (2018). Study on the effects of an oral lactobacilli and lactoferrin complex in women with intermediate vaginal microbiota. Arch Gynecol Obstet.

